# The development of telemedicine programs in Sub-Saharan Africa: Progress and associated challenges

**DOI:** 10.1007/s12553-021-00626-7

**Published:** 2021-11-25

**Authors:** Joana Eva Dodoo, Hosam Al-Samarraie, Ahmed Alsswey

**Affiliations:** 1 College of Distance Education, Department of Business Studies, University of Cape Coast, Cape Coast, Ghana; 2 School of Design, University of Leeds, Leeds, UK; 3 Centre for Instructional Technology & Multimedia, Universiti Sains Malaysia, Penang, Malaysia; 4 Department of Multimedia Technology, AL-Zaytoonah University of Jordan, Amman, Jordan

**Keywords:** Telemedicine, Sub-Saharan Africa, Healthcare readiness

## Abstract

Monitoring the progress of telemedicine use in Sub-Saharan Africa (SSA) countries has received a considerable attention from many health organizations and governmental agencies. This study reviewed the current progress and challenges in relation to the development of telemedicine programs in SSA. The results from reviewing 66 empirical studies revealed an unbalanced progress across SSA countries. Further, technological, organisational, legal and regulatory, individual, financial, and cultural aspects were identified as the major barriers to the success of telemedicine development in SSA. This study reported the current trends in telemedicine application, as well as highlighting critical barriers for consideration by healthcare decision makers. The outcomes from this study offer a number of recommendations to support wider implementation and sustainable usage of telemedicine in SSA.

## Introduction

The use of telemedicine systems in healthcare provision has received wide endorsement from the policy makers, medical practitioners and researchers. Monitoring the developing of telemedicine programs has received a wide attention recently in most developed countries. This can be due to the effectiveness of telemedicine in facilitating rapid access to specialists, diagnosis, treatment, and prevention of injuries and diseases [1, 2]. It is useful particularly in emergency response situations such as the recent COVID-19 pandemic [3]. Telemedicine was instrumental in the fighting of major epidemic outbreaks such as the severe respiratory syndrome epidemic (SARS) in Taiwan, H1N1 and H7N9 pandemic influenza in China, and Middle-East respiratory syndrome (MERS) coronavirus epidemic [4]. Behar, et al. [5] reviewed the global uptake of telemedicine in the wake of the COVID-19 global pandemic. The authors demonstrated how various countries across the globe have deployed telemedicine as an efficient route for testing, remote monitoring of patients with mild COVID-19 symptoms, contact tracing and symptom triage.

Telemedicine programs are not new to healthcare organizations in SSA. These programs have supported the strengthening of healthcare, patient and professional health-related education, surveillance and prevention of diseases in SSA [6]. They were effective in the fighting of the Ebola virus disease in some parts of Africa [4]. Recent studies suggest a renewed effort towards the implementation of telemedicine systems in SSA, in particular as an alternative healthcare route in the era of COVID-19 crises [5, 7]. This was possible due to the wide usage of mobile telecommunication systems in SSA countries. Holst, et al. [8] commented that telemedicine systems can be a potential gamechanger in healthcare provision in SSA. According to them, 41 countries in SSA have in place national digital health strategies and architectures (NDHSA). It is projected that the mobile telephone connections of 816 million as at 2019 in SSA will increase to 1.05 billion by 2025 [9], thereby making it easier for a wider use of telemedicine applications.

Although the above studies have shown how various programs of telemedicine can support the effort to curtail the negative impact of COVID-19 in SSA, they do not indicate the progress and the extent made with regard to the implementation of telemedicine in these countries. For example, Edoh, et al. [10] attempted to review telemedicine system user satisfaction in two West African countries. The authors reported a major lack of studies on telemedicine progress to serve as a baseline for indicating users’ satisfaction with telemedicine services. Adebayo, et al. [11] limited their study to tele-neurology practice and infrastructural preparedness in SSA, which limits the extent to which various countries in SSA have made progress in terms of telemedicine implementation.

In addition, previous literature has attempted to describe telemedicine uptake in a piecemeal manner or limited to specific telemedicine services, whereas differences were reported among SSA countries. This gap presents an opportunity to investigate progress in the development of telemedicine programs and associated challenges in SSA. It is anticipated that the outcomes from this review will offer timely directions to policy makers on the extent of telemedicine implementation for strategic regional collaborations. It will also determine the various phases of telemedicine implementation that can potentially assist in addressing the perceived repetitions of certain telemedicine systems observed in SSA countries such as South Africa [6, 12]. This study is important in that it offers a holistic view of the extent of telemedicine progress and offers a pathway for continuity and advancement of telemedicine systems in SSA.

## Methodology

This study was designed to answer two research questions: “What is the current progress in the use and adoption of telemedicine programs in SSA countries?” and “What are the associated challenges of implementing telemedicine programs in these countries?” We adapted the Preferred Reporting Items for Systematic Reviews and Meta-Analyses (PRISMA) guidelines [13] to answer these questions. PRISMA was used in this study because it offers detailed and step-by-step instructions for identifying and evaluating previous studies in relation to the current research area [14, 15].

### Articles search and inclusion criteria

This study was conducted to provide a roadmap for healthcare decision makers to understand the current development of telemedicine programs in SSA. We used quotation marks in the search process in order to produce the desired intersection. Key words used include: (“progress” OR “factors”) AND (“affect’ OR “impact” OR “influence” OR “utilized” OR “adopt” OR “implement”) AND (“telemedicine” OR “e-health” OR “telehealth” OR “eHealth readiness” OR “electronic health” OR “electronic health record”) AND (“Sub-Saharan African countries” OR “African countries” OR “SSA countries” OR “Community of Sahel-Saharan States” OR “Economic Community of West African States” OR “Economic Community of Central African States” OR “East African Community” OR “Southern African Development Community” OR “the Country name”). The retrieved papers were peer-reviewed and published in journals, conference proceedings/chapters, or university repositories. During our search of the literature, there were no restrictions placed on the language, data, or publication status of the articles. We only included empirical studies and governmental reports. The date of publication was not limited in this review.

### Screening and coding of the articles

**Fig. 1:**
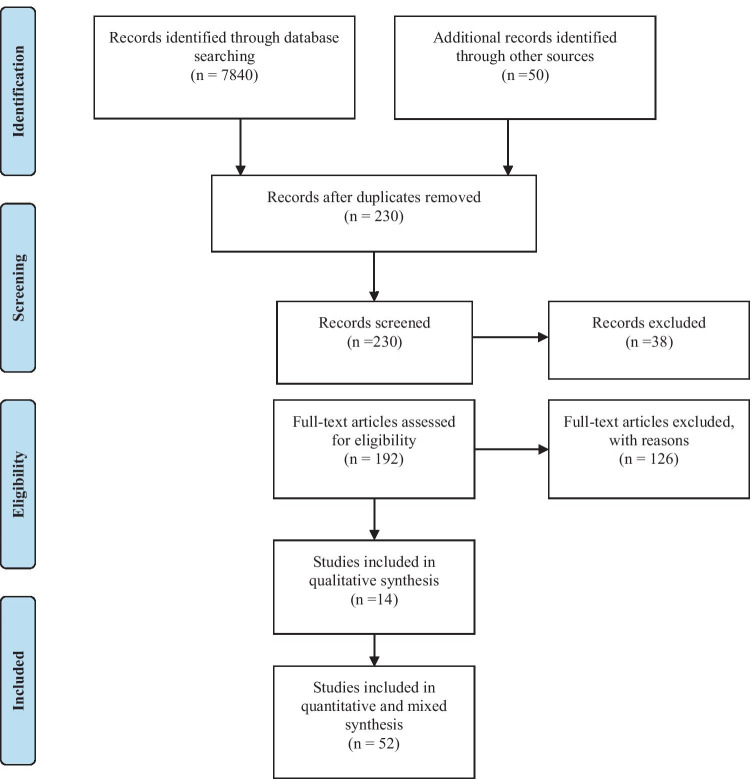
Study selection flowchart

We were able to identify/filter the relevant and non-relevant studies for this review. We used specific keywords to retrieve articles that can be used to answer the research questions. A total of 7840 studies dies were retrieved from multiple databases such as PubMed, IEEE Xplore Digital Library, Cochrane, ProQuest, Web of Science, Scopus, Global Health, and Google Scholar. A total of 230 studies were duplicates (which we removed). Further, we screened all the titles and abstracts related to the search keywords. After applying the inclusion criteria discussed above, a total of 192 studies were finalized for full text review. This led to further exclusion of 126 articles, either because the articles covered theoretical and conceptual aspects. The finalized 66 studies were then subjected to full text review to examine the progress and challenges of telemedicine programs in SSA countries. The identified challenges (based on the country where the research was conducted) were placed into six categories/dimensions: organisational, technological, financial, individual, culture, and legal barriers. Challenges identified in the final list of articles were categorized using an item-focused coding approach due to its heterogeneity across disciplines. The barriers were placed under the technological dimension if they were related to information system and connectivity software. The barriers that were placed under the financial dimension were those related to financial resource and operational cost of telemedicine. The barriers that were placed under the individual dimension were related to users’ experience, knowledge, and awareness of telemedicine systems. The barriers related to certain social constraints and traditional beliefs inside the community were placed under the cultural dimension. Lastly, the barriers that were related to the policies, legislation, standard governing confidentiality and privacy for the use of telemedicine were placed under the legal and regulatory dimension. As for the quality check**,** two experts were assigned to evaluate the final list of articles. Both experts used a spreadsheet to compile their recommendations on whether the article was relevant to this study, and a consensus meeting was called to exchange their observations. The interrater reliability value was calculated using an item-by-item method, specifically by dividing the tally of agreements by the total number of agreements and disagreements, divided by 100 [16]. The average value for the interrater agreement was 86% (Fig. 1).

## Progress in telemedicine in SSA

**Fig. 2:**
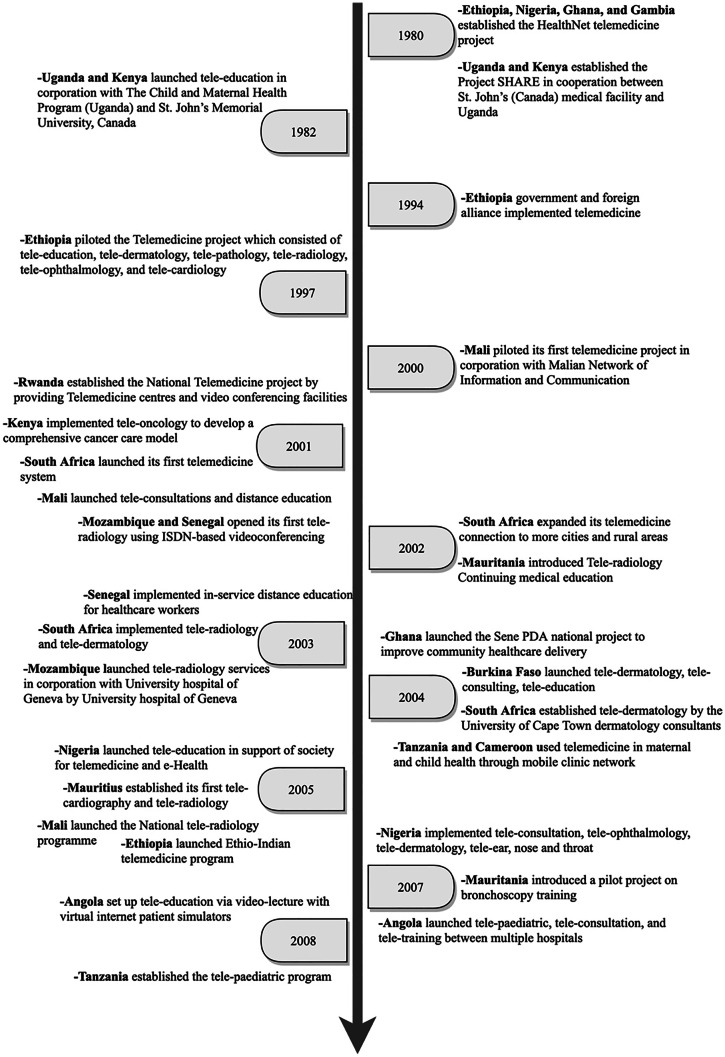
Progress in telemedicine use in SSA countries [1980 – 2008]

**Fig. 3:**
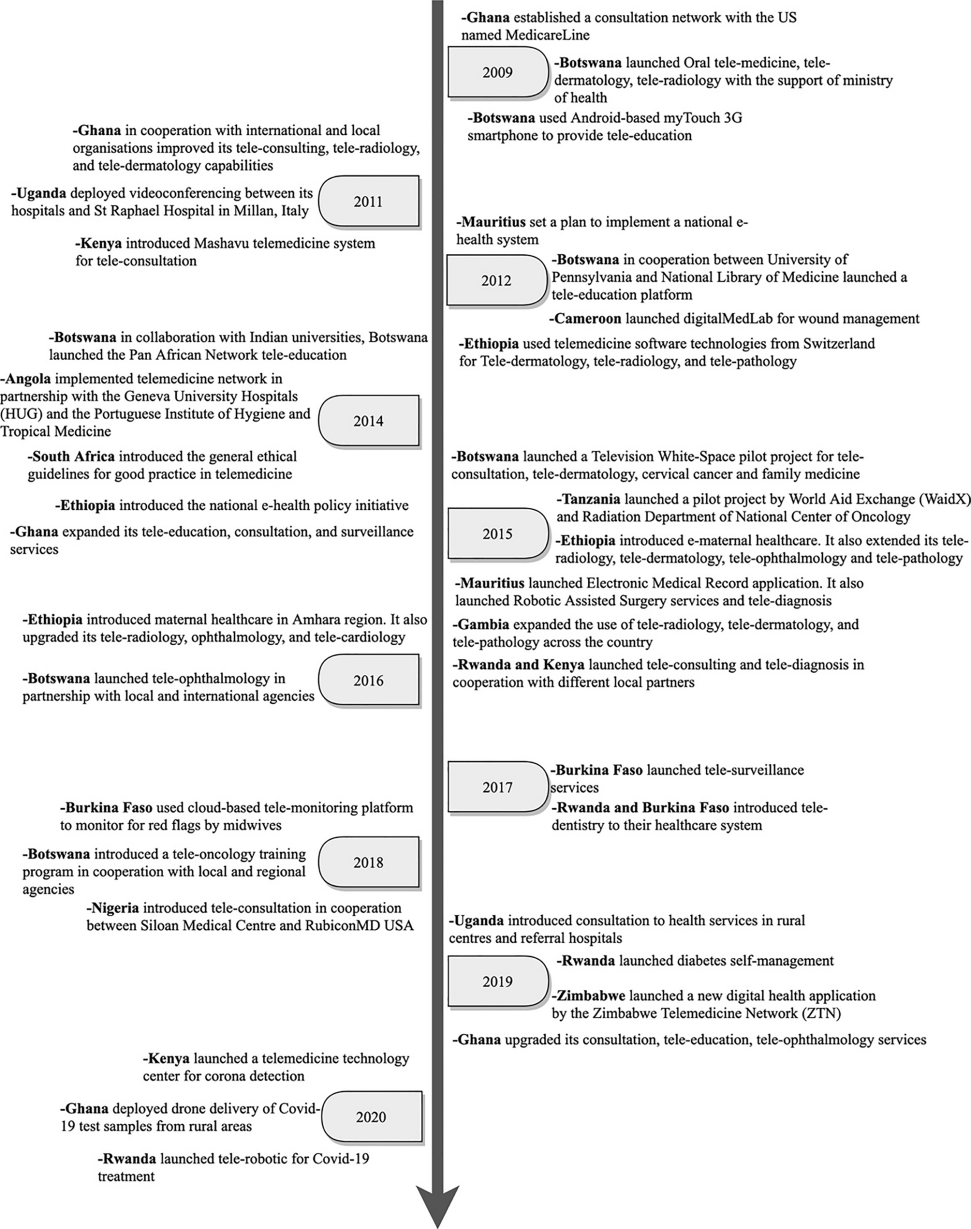
Progress in telemedicine use in SSA countries [2009 – 2020]

Our review of the literature (see Fig. 2–3 and Appendix) showed a number of attempts by individual governments, international partners and health organisations to develop the capacity of SSA countries to effectively execute telemedicine systems. In order to represent progress of telemedicine, we followed a similar trend by Dodoo, et al. [6] for the presentation of our results. Based on the African Union’s regional economic groupings, the following key regions were emerged from the review of the literature: Community of Sahel-Saharan States (CSSS), Economic Community of West African States (ECOWAS), East African Community (EAC), South African Development Community (SADC) (https://au.int/en/organs/recs). The findings were categorised based on the existing regional segregation as follows:

### Community of Sahel-Saharan States (CSS)

The early uptake of telemedicine in this region began in Ethiopia in 1980 under the HealthNet project. Between 1994 and 1997 Ethiopia established the Ethiopian Telecommunications Corporation (ETC) which recognized telemedicine as one of its services. However, these efforts were limited to planning and establishing the basis for piloting telemedicine in the region. Furthermore, between 2011 and 2012, through the Gezira Family Medicine Project in collaboration with the Faculty of Medicine, University of Gezira and the Ministry of Health, Sudan implemented a telemedicine program. Variants of telemedicine systems implemented include internal medicine, tele-paediatrics, dermatology, obstetrics and gynaecology, and e-learning via video conferencing.

Previous studies suggested that the implementation of telemedicine programs in this region has not seen much progress due to the lack of a framework for balancing multiple stakeholder interest. According to Kifle, et al. [17], most of the telemedicine projects faced various sustainability-related issues, especially after the completion of the project period. Also, a number of telemedicine initiatives were mostly funded from external support and lacked strategies for continuity and support from key stakeholders (Government, private sector, non-governmental organisations and the larger community) [18]. The failure to reach out to key stakeholders other than the healthcare sector may serve as a major challenge, especially in the area of system maintenance policy. Another key concern was the impact of telemedicine on existing work-structure. We identified that most healthcare professionals in the region face enormous workload. This led healthcare professional to view telemedicine system as an additional task that would result in increased workload and time consuming [19, 20]. These factors serve as a vital challenge and can potentially constrain the progress of telemedicine projects.

### Economic community of West African States (ECOWAS)

Most of the early telemedicine projects started in countries such as Nigeria, Ghana, and Gambia in 1980 under the HealthNet project. Between 2000–2001 Mali was the first to successfully pilot a telemedicine program in cooperation with the Malian network of information and communication. The piloted project was mainly aiming at providing internet-based technologies for distance learning and teleconsultations. Other countries, such as Senegal, followed suit with the same measures to implement telemedicine using videoconferencing methods with a focus on patient care. Between 2003 and 2007, more improvements in the use of telemedicine systems have been reported across Ghana, Nigeria, and neighbouring countries. This includes using telemedicine for cancer care and education. In addition, the use of teleradiology, telepathology, and tele-oncology was initially introduced in these countries in an attempt to increase the reach of these services to rural areas. For example, Ghana in 2009 established a consultation network with Medicare line (US) to facilitate the communication between physicians and surgeons using free calls and SMS messages. In 2017, the governments of some countries like Burkina Faso, and Nigeria launched initiatives to integrate cloud computing for building their telemedicine infrastructure. This includes providing surveillance of certain patients and spread of disease, as well as advancing capacity of teleconsultation and monitoring services in these countries. In 2019, Ghana focused on upgrading the current telemedicine services to reach their full potential in smaller rural communities. Recently, the utilization of telemedicine services in this region has increased dramatically due to various challenges associated with COVID-19 and the need for continued healthcare provision. Ghana has also shown progress in using telemedicine by deploying drone delivery of COVID-19 test samples from rural areas.

### East African community (EAC)

Kenya and Uganda introduced a telemedicine project in cooperation with several international organizations from 1980 till 1982 to plan for technology implementation. Other countries, such as Rwanda, Kenya, followed suit with the same measures to ensure effective use of telemedicine using videoconferencing methods with a focus on patient care. Between 2003 and 2007, more improvements in the use of telemedicine systems have been reported across Tanzania, and neighbouring countries for cancer care and education. In addition, there was expansion of telemedicine services to include teleradiology, telepathology, and tele-oncology in order to increase the reach of these services to underserved communities. Between 2007 and 2008, Tanzania was successful in introducing tele-paediatrics to increase their capacity for delivering specialist paediatric and child healthcare services. Another major development was in the involvement of international corporations, as a key source of technology transfer in the region.

In addition, the increasing popularity of e-health and m-health technologies, especially between 2012–2016, has led to a rapid growth of telemedicine services in the region. In 2019, countries like Uganda, Rwanda, Zimbabwe, have focused on upgrading the current telemedicine services to reach their full potential in smaller rural communities. For example, Rwanda launched a new diabetes self-management centre to help provide care and monitor patients in rural areas. This includes the launch of telemedicine centre to assist hospitals in detecting COVID-19 cases in Kenya. Rwanda introduced tele-robotics to treat COVID-19 cases. Uganda witnessed an increased use of teleconsultation to ensure continuity of healthcare for patients, established call centres and online health to provide triage and referral services to the general public. In addition, mobile SMS services were employed widely to spread awareness about COVID-19 infection and prevention measures.

### Southern African development community (SADC)

A successful attempt to use telemedicine services (mainly internet-based technologies) was achieved by South Africa and Mozambique for distance learning and teleconsultations between 2000–2001. A further development in the utilization of telemedicine was noted between 2002 and 2003 through the expansion of care services to rural and urban areas of South Africa. Between 2003 and 2007, more improvements in the use of telemedicine systems have been reported across the country. This includes using telemedicine for cancer care and education. In addition, the use of teleradiology, telepathology, and tele-oncology was introduced in SADC countries to expand the range of their telemedicine services.

Between 2007 and 2008, Angola was successful in introducing tele-paediatrics to expand specialist paediatric and child healthcare services. These efforts at health technology transfer in the region were successful in partnership with international corporations. For example, the current 3G network Smartphone equipped with data in Botswana enhances faster health information transmission, quicker medical response and improved patient outcomes. This has advanced the growth of telemedicine program in the region by offering conversational tele-education and teleconsultation services. Also, Mauritius and Botswana have implemented a nationwide telehealth project in an attempt to promote a wider access for patient care while advancing medical education for physicians by providing comprehensive active clinical programs, virtual educational programs, and electronic libraries. In 2017, the governments of Botswana launched initiatives to integrate cloud computing for building their telemedicine infrastructure. This includes providing surveillance of certain patients and spread of disease, as well as advancing the capacity of teleconsultation and monitoring services in these countries. The negative impact of COVID-19 and the potential spread of the virus through face-to-face hospital attendance influenced the Health Professions Council of South Africa to revise restrictions on the current telemedicine practices. This represents a breakthrough in terms of progress and acceptance of telemedicine implementation.

## Implications for healthcare practitioners and providers

This study reported the progress in relation to the development of telemedicine programs in specific regions in SSA. Our review of the literature (see Appendix) suggests that a reasonable progress has been made to effectively use telemedicine in SSA, in particular during the COVID-19 period. Yet, many knowledge gaps still remain in terms of the extent of usage and suitability. This study shed the light on progress made by individual countries/regions in SSA to implement telemedicine.

We found variations in terms of extent of telemedicine applications across SSA countries. Most of the telemedicine systems were implemented on a pilot project basis. For example, aside from the initial telemedicine start-up projects, in the community of Sahel Saharan States (particularly Ethiopia), the existing literature pointed to less progress in implementing and expanding telemedicine services. This finding provides policy directions for stakeholders to invest in digital health innovations to help address challenges related to accessing healthcare provision in the region.

In addition, our findings showed how a range of telemedicine services can be implemented in the regions of ECOWAS, EAC and SADC, thus signifying some level of progress in these regions. The study’s findings may potentially pave the way for scaling up telemedicine services. For example, the results show that tele-education can be used extensively for medical education in most SSA countries. This may potentially offer an effective way for strategic regional collaboration that is useful for training medical personnel. It is particularly significant for facilitating exchange programmes among medical students in SSA. In addition, it will facilitate some level of parity in the development of human resource for telemedicine practices. The effective use of tele-education can address the challenges associated with the lack of telemedicine expertise among clinicians, ethical and privacy concerns, which were identified as barriers to the successful implementation of telemedicine systems [6]. Aside from these, tele-education has implication for promoting mobility of medical personnel across regions and countries in SSA.

Another issue worthy of attention was related to the use of the store-and-forward method in most of the telemedicine services. By this method, patients’ medical information is sent for expert review via email, messages or the eHealth system. However, the store-and-forward method has been reported to be deficient in generating user satisfaction of telemedicine due to the lack of a real-time experience. We, therefore, recommend policy makers to include video-conferencing and other advanced methods to enhance the quality of services and continued usage of the system. This can potentially improve users’ perception about digital health technology and services, thus leading to sustainability and value creation. We recommend for further research to document initiatives on digital health technology usage and empirically assess their impact on healthcare practice in SSA.

## Conclusion

This study traced the implementation of telemedicine programs in SSA and grouped progress according to the regional categorisation. Extensive use of tele-education services in the training of medical students was reported across SSA. This can promote regional collaboration across SSA by increasing standardisation of telemedicine practices. The findings from the literature review suggests that the implementation of telemedicine systems in the Sahel-Saharan States has not seen much progress in terms of expansion of services following the completion of the initial projects. Further, our review suggests that reasonable progress has been made in the ECOWAS and EAC regions in terms of implementation, expansion and sustainability of telemedicine systems and services, especially during the COVID-19 period. The SADC has been successful in implementing a broad range of telemedicine systems and services, which is backed by strong national legislations to support its sustainability. Based on the above, the unbalanced implementation of telemedicine systems calls for urgent stakeholder attention to improve the current healthcare provision for increased user acceptance and satisfaction.

## Appendix

Table 1

**Table:**

References	Regions/ Countries	Service collaborators	Telemedicine service	Telemedicine systems
	CSSS			
Mbarika [21]; Kifle et al. [22]; Lemma et al. [23]; Kifle et al. [17]; Shiferaw and Zolfo [24]; Hailemariam et al. [18]; Abera et al. [25]; Medhanyie et al. [19]; Xue et al. [26];Shiferaw et al. [27]; Weldegebrial and Berhie [28]	Ethiopia	HealthNet telemedicine project, Addis Ababa University (AAU), British Council Telecenter, the International Telecommunication Union, WHO, ORBIS Ministry of Health, ETC, UNECA, UNESCO, United Naions Economic Commission for Africa, infoDev	Tele education, tele dermatology, telepathology, teleradiology, tele ophthalmology, telecardiology, e-maternal health care;	Dial up internet connection, LAN; Personal computer, digital cameras, telemedicine software; Store-and-forward; WDS technologies from Switzerland; remote patient monitoring
Colt, et al. [29]; Bagayokoa, et al. [30]	Mauritania	Pilot project with the World Bronschology Foundation and Nauadhibou Regional Hospital	Tele education, Teleradiology Continuing medical education,	Web-based instruction
	ECOWAS			
Mbarika [21]; Adebola and Lawson [31]; Mars [32]; Godstime et al. [33]; Olayiwola, et al. [34]	Nigeria	HealthNet telemedicine project; Society for Telemedicine and e-Health in Nigeria (SFTeHIN), WHO Telemedicine centres, Norway Telemedicine centre, National Information Technology Development Agency, Pan African e-Network clinical telemedicine project, Ibadan Super Specialty hospital, California Hospital, USA Siloan Medical Centre in partnership with RubiconMD USA	Medical alerts, Tele education, Teleconsultation, teleophthalmology, tele dermatology, tele-ear, nose and throat, teleconsultation	Video conferencing, Image & illumination system, cameras, electronic stethoscope
Mbarika [21]; Kennedy et al. [35]; Luk et al. [36]; Mars [32]; Yusif and Jeffrey [37]; Nyame-Asiamah [38]; Tchao et al. [39](2019); https://www.novartisfoundation.org/our-work/reimagining-healthcare-through-digital-technology/ghana-telemedicine; https://allafrica.com/stories/202004300181.html; https://www.voanews.com/COVID-19-pandemic/COVID-19-drives-health-care-tech-innovation-ghana	Ghana	HealthNet telemdicine project, London School of Hygiene and Tropical Medicine, Geneva, Korle Bu hospital (Ghana) and Moorfields eye hospital London, Ghana Consultation Network and US, MedicareLine (Ghana), Pan African e-Network clinical telemedicine project, Novartis Foundation, Millenium Promise Alliance, Earth Institute, Columbia University, St. Martin’s Hospital, MedGate, Ericsson and Airtel, Sandford US based (2012), Family health hospital in collaboration with Family Health Group Apollo Hospital (India), eNetwork, Zipline drone-delivery, health care tech company Redbird (rapid testing), Talamus Health Ghana company	Research, surveillance, Teleophthalmology, Continuous education, e-consultation, Telemedicine education, teleradiology, tele dermatology, COVID-19 testing, COVID-19 healthcare (diagnose, monitor infections)	Web-based telemedicine application, asynchronously synchronized, Video, store-and-forward conferencing Mobile application, video appointment
Caumes et al. [40]; Jacq et al. [41]; Diallo et al. [42]; Arnaert et al. [43]	Burkina Faso	Physicians in Burkina Faso and France (Clermont-Ferrand), Developed by Davycas International and Financed by the MenAfriNet Consortium, BELT framework, funded through Grand Challenges Canada	Tele dermatology, teleconsulting, tele education, Diabetic retinopathy, Tele-surveillance, Antenatal healthcare, Nationwide cloud-based meningitis surveillance and specimen tracking system (STELAB) for real-time data reporting	point-and-shoot digital camera equipped with a Carl Zeiss Vario Sonnar lens and a flash, Grid technologies Healthgrid (stores data of medical interest for easy access in healthcare), Barcodes for tracking, online-offline application, cloud-based telemonitoring platform (used to monitor for red flags by midwives)
Mbarika [21]; Kennedy, et al. [35]; http://origin.who.int/goe/publications/atlas/2015/gmb.pdf; http://www.aiu.edu.gm/aiu_telemedicine.html	Gambia	HealthNet telemedicine project, Royal Victoria Teaching Hospital (Gambia) with Moorfields eye hospital London, AIUWA (American International University West Africa) Telemedicine centre, partners African Union, Government of The Gambia and Pan African eNetwork project (India)	Teleophthalmology, Teleradiology, tele dermatology, telepathology, Tele education	Web-based telemedicine application
Mbarika and Okoli [44]; Geissbuhler et al. [45]	Senegal	SONATEL (telecommunication operator in Senegal), Lille Regional University hospital and the European Institute of Telemedicine in Toulouse partnership	In-service distance education for healthcare workers, Tele-cardiology and tele-neurology, tele-echography	Teleconferencing, store-and-forward method of information transmission, ISDN-based Visio conference
Geissbuhler et al. [45]; Mars [32]; Bagayokoa et al. [30]; Sangare et al. (2015)	Mali	National Telemedicine Project in Mali partnership with Mali University Medical School and Geneva University Hospital. First telemedicine pilot project by Malian Network of Information and Communication, funded by State of Geneva, RAFT (Network in French speaking Africa for Telemedicine)	Teleconsultations and distance education, Teleradiology Continuing medical education	IKON hardware and software X-ray and mammogram images, IKON platform
	CAC			
Katzenstein et al. [46]; Kingue et al. [47]; https://www.movementdisorders.org/MDS/Regional-Sections/Sub-Saharan-Africa/Telemedicine-in-Douala-Cameroon-.htm; https://www.idgconnect.com/idgconnect/interviews/1028321/cameroon-telemedicine-approach-wound-management	Cameroon	Movement Disorder Society Telemedicine taskforce and Hospital Laquintinie in Douala Labotel (NGO), telemedicine association of Cameroon and Ministry of Public Health, digitalMedLab (Zurich), Switzerland by Cameroonian physician (Dr. Patricia Sigam) and Australian developer, Telemed-cam project partnered by Telemedicine centre at Yoounde General Hospital and rural health centres	Tele education, Maternal health; Wound management; Telecardiology	WoundDesk Mobile app Telemedcam mobile app, real-time and asynchronous interaction
	EAC			X-ray and mammogram images, IKON platform
Mbarika and Okoli [44]; Qin et al. [48]; Ferrari et al. [49]; https://www.ncbi.nlm.nih.gov/pmc/articles/PMC1492060/pdf/cmaj00136-0088.pdf; https://www.scidev.net/sub-saharan-africa/medicine/news/kenya-launches-telemedicine-initiative-poor.html?; https://www.axisimagingnews.com/news/kenya-opens-first-telemedicine-center-for-COVID-19-detection	Kenya	International Satellite Organisation and Project SHARE. St. John’s (Canada) medical facility and Nairobi medical faculties, Canadian International Development Agency (CIDA), AMPATH-Oncology with North America, Mashavu Telemedicine system, National telemedicine project, Kenyatta National Hospital, Kenya Ministry of Health and Merck Group Germany, Telemedicine technology center for corona detection	Tele-ECG, Enhanced medical education, Tele education, Tele oncology, Teleconsultation, telediagnosis, tele detection	Video conferencing, VIA (Visual inspection with acetic acid, store-and-forward system,
Mbarika and Okoli [44]; https://www.ncbi.nlm.nih.gov/pmc/articles/PMC1492060/pdf/cmaj00136-0088.pdf	Uganda	International Satellite Organisation and Project SHARE, St. John’s (Canada) medical facility and Uganda, The Child and Maternal Health Program (Uganda), Canadian International Development Agency (CIDA), Pan African e-Network clinical telemedicine continuing telemedicine project and Makerere University, Ugandan hospitals and St Raphael Hospital in Millan, Italy Hospitals in Kampala. Launch of Coolscope at Mulago Hospital Ugandan health services in rural centres and referral Hospitals	Tele-ECG, Teleeducation Teleconsulting, Telepathology,	
https://allafrica.com/stories/201905130465.html	Zimbabwe	Zimbabwe Telemedicine Network		Videoconferencing, store-and-forward
Kennedy et al. [35]; Katzenstein et al. [46]; Krüger and Niemi [50]; Balogun et al. [51];	Tanzania	CCBRT Disability hospital (Tanzania) with Moorfields eye hospital London, Mission Mikocheni Hospital (Tanzania), HealthSpan International foundation (USA), Tanzanian Telemedicine Network with open-source software iPath Pilot project by World Aid Exchange (WaidX) and Radiation Department of National Center of Oncology, Yerevan, Armenia	Teleophtalmology, Maternal and child health, Telepaediatric, Teleoncology,	Web-based telemedicine application mobile clinic network Store-and-forward via Videoconferencing
Nchise et al. [52]; Murererehe et al. [53]; Kabeza et al. [54]; https://spidercenter.org/files/2017/07/Report-Telehealth-Rwanda-100710-Web.pdf; https://allafrica.com/stories/202005140352.html	Rwanda	National Telemedicine project and Rwanda Ministry of Health, Ministry of IT Authority and Partners from Belgian Technology operation, Pan-African E-network, SMART Rwanda master plan, eC3-electro cervical cancer control program, Kigali Hospital, Rwanda’s vision 2020 agenda, Open Medical Record System (OpenMRS), TRACnet and TRACPlus and HMIS health intervention projects, First Rwanda diabetes self-management application (Kir’App), CRUZR robots, manufactured by Kigali-based Belgian robotic tech firm (Zorabots), Rwanda Biomedical Centre, Rwanda Information Society Authority and Rwanda Utilities Regulatory Authority	Telemedical training, Teleconsulting and telediagnosis, Tele dentistry, Diabetes self-management, Tele-robotic for COVID-19 treatment	video conferencing facilities, PAN-African e-network satellite, LMS, Humanoid robots with video conferencing capacity to connect to doctor, ‘Kir’App’ via smartphone
Bholah and Beharee [55]; Beebeejaun and Chittoo [56]; Ratna et al. [57]; https://mauritiusclinics.com/en/our-services/our-medical-services/medical-services/telemedicine	Mauritius	The SAFE project, Clinique du Nord with Manipal Hospital in India; e-Health start-up project in collaboration with the Mauritius Research Council and State Informatics Limited, Appollo Bramwell Hospital	Tele cardiography and teleradiology; Teleconsultation, Robotic Assisted Surgery services, telediagnosis	Electronic Medical Record application, “Da Vinci System” features magnified 3D high-definition vision system
Mbarika and Okoli [44]; Mbarika [21]; Geissbuhler et al. [45]; Colt et al. [29],	Mozambique	University hospital of Geneva, The Telecommunications Development Bureau of the International Telecommunications Union, Central hospitals of Beira and Maputo, SATELLIFE, A demonstration project between (FISSA) Dakar and Mozambique, Pilot project on bronschoscopy training with grant from the World Bronschology Foundation and Maputo Central Hospital	Teleradiology Teleeducation	Digital microwave transmission, terrestrial satellite telecommunication systems, WDS Technology, standard low-cost teleradiology equipment, ISDN-based Visio conferencing, Web-based instruction
	SADC		Teleradiology Tele education	
Gulube and Wynchank [58]; Fortuin and Molefi [12]; Mars [59]; Kennedy et al. [35]; Mars [32]; Colven et al. [60]; Webster [7]; http://www.kznhealth.gov.za/telemedicine1.pdf; http://www.kznhealth.gov.za/telemedicine1.pdf; https://www.hoganlovells.com/en/publications/the-regulation-of-telemedicine-in-south-africa	*South Africa*	National Telemedicine system (launched pilot project phase I, Mindset Health channel with National Department of Health, Mindset and Sentech, the Medical Research Council of South Africa, PHASE II – effective telemedicine connection, PHASE III additional and affordable telemedicine healthcare (Transfer from pilot stage to clinical and operational stage), Groote Schuur Hospital (Cape Town,SA) in partnership with Moorfields eye hospital London	Teleradiology, tele-ultrasound (antenatal services), telepathology and teleophthalmology, tele dermatology, programme in medical education	ISDN at 256 kbit/s, Store-and-forward, satellite, computer, television screen, Web-based telemedicine application, Video conferencing, CT scanners, store-and-forward technology
Littman-Quinn et al. [61]; Armstrong et al. [62]; Chang et al. [63]; Munyoka [64]; Chavez et al. [65]; Chandar et al. [66]; https://www.odess.io/files/documents/enquetes/CR_ODESS2017-Peek_English.pdf	*Botswana*	Botswana-UPenn Partnership, The Ministry of Health of Botswana, Clinton Health Access Initiative, University of Botswana, the National Library of Medicine, Positive Innovation for the Next Generation (PING), Hewlett-Parkard, Orange Foundation and Mascom Wireess, University of Botswana School of Medicine (SOM) and Resident Physicians (remote areas), Pan African Network Tele-education projecct to support distance education, Television White-Space pilot project (Project Kgolagano), Innovation Hub Microsoft, Vista Life Sciences, Global Broadband Solution, Standard Chartered Bank, Rugers-Cancer Institute of New Jersey (R-CINJ) fellowship program, partnerships with Rutgers Global Health and University of Botswana	Oral telemedicine, Teledermatology, teleradiology, Teleducation, Teleconsultation, cervical and family medicine, Teleophthamology, Teleoncology training	mobile technology, Android-based myTouch 3G smartphone equipped with data-enabled subscriber identity module and built-in camera, e-mails/Web access, Tele-education Learning Management System (IP-based and VSAT networks), TV White Space technology, Potable Eye Examination Kit (Peek vision technology)
Dinis et al. [67]; Bediang et al. [68]; Zennaro et al. [69]; Correia et al. [70]	Angola	PEDITEL project, Europe-Africa Telemedicine network, collaboration between hospitals in Angola and Portugal, Angola Telecom, Multitel, Unitel RAFT project Hospital Divina Providencia and IRCCS Burlo project Angolan National Telemedicine Network in partnership with the Geneva University Hospital and Portuguese Institute of hygience and Tropical Medicine, RAFT network	Telepediatric, teleconsultation, tele-training Teleducation Teleradiology	videoconferencing, real-time teleconsulting, ISDN lines Video-lecture with Dudal, virtual internet patient simulators (VIPS) X-ray photosensitive screens
